# Application of a novel scoring system for gastric cancer opportunistic screening in hospital visits

**DOI:** 10.1186/s12876-022-02315-9

**Published:** 2022-05-08

**Authors:** Qingjie Zhou, Yihan Chen, Jie Pan, Leying Zhou, Jiejun Lin

**Affiliations:** Department of Gastroenterology, Wenzhou Central Hospital, Wenzhou, 325000 Zhejiang China

**Keywords:** Novel scoring system for gastric cancer screening, Gastric cancer opportunistic screening, Gastric cancer, Early gastric cancer, Precursors of gastric cancer, Hospital visit

## Abstract

**Background:**

A novel scoring system and screening procedure for gastric cancer (GC) screening was proposed based on the national conditions of China, which state that endoscopy professionals and facilities are relatively limited compared with the large Chinese population.

**Methods:**

A novel scoring system for gastric cancer screening was used to retrospectively analyse the patients who met the screening procedure from April 2017 to December 2019 in our hospital. We divided all of the patients into three groups: low-risk group (0–11 scores), medium-risk group (12–16 scores), and high-risk group (17–23 scores). Statistical analysis was performed on the detection rate of gastric cancer and precursors of gastric cancer among these three groups.

**Results:**

A total of 6701 patients were enrolled in this study, including 4,352(64.95%) in the low-risk group, 1,948 patients (29.07%) in the medium-risk group, and 401 patients (5.98%) in the high-risk group. The total detection rate of gastric cancer was 2.84% (190/6,701), with a 0.94% rate (41/4,352) in the low-risk group, a 5.18% rate (101/1,948) in the medium-risk group, and a 11.97% rate (48/401) in the high-risk group. There were statistically significant differences in the detection rate of gastric cancer among these three groups (all *P* < 0.05). The detection rate of early gastric cancer was 46.31% (88/190) among all of the detected gastric cancers in this study. In addition, the detection rates of differentiated gastric cancer and precursors of gastric cancer in the medium-risk group and high-risk group were significantly higher than those in the low-risk group. In addition, the area under the curve (AUC) of the receiver operating characteristic curve (ROC) of the novel scoring system in differentiating GC was 0.79.

**Conclusion:**

The screening strategy based on the novel scoring system can significantly improve the efficiency of gastric cancer opportunistic screening in hospital visits. Gastroscopy should be strongly recommended for patients in the medium-risk group and high-risk group, and detailed gastroscopy should be adopted as early as possible to improve the detection rate of early gastric cancer.

## Introduction

Gastric cancer is the sixth most common cause of new cancer cases and the third most common cause of death among annual incidences of cancer throughout the world, according to the global cancer statistics presented in 2020 [1, 2]. Additionally, its incidence is particularly high in East Asia, including China, Japan, and South Korea [3–5]. Although the incidence of GC has been declining in recent years, China still has the most GC patients throughout the world [6–8]. The incidence and mortality rates of GC are relatively high in China and almost 500,000 persons ultimately die of GC every year in China, which accounts for approximately half of the worldwide cases and deaths [9–11].The prognosis of GC is closely related to its stages. For example, the prognosis of advanced gastric cancer (AGC) is poor and its 5-year survival rate is only approximately 30%,; however, the rate could be above 90% for early gastric cancer (EGC) after treatment [12, 13]. Therefore, a timely and accurate diagnosis is important for the treatment and prognosis of patients with GC [14]. However, EGC is difficult to diagnose, due to patients being either asymptomatic or having nonspecific symptoms. Most GC cases are diagnosed at an advanced stage, and the detection rate of EGC in China is less than 10% (on average) [15]. With the improvement of endoscopic techniques and the awareness of endoscopic professionals, the detection rate of EGC has improved in recent years, but it is still significantly lower than that in Japan (70%) or South Korea (50%) [7, 16].

In Japan and South Korea, gastroscopy is readily available and affordable and has become a major screening tool recommended for men and women aged 40 years and older [17, 18]. However, it is unlikely that screening gastroscopy can be offered to all populations in China because endoscopy professionals and facilities are relatively limited compared with the large Chinese population [19, 20]. Therefore, it is important to develop a prediction rule for estimating GC risk and an appropriate GC screening procedure for the Chinese high-risk population. In view of this, a novel scoring system and screening procedure for GC screening (the Expert consensus on Screening procedure of EGC in China) was proposed in December 2017, with an aim to establish an EGC screening procedure suitable for China's national conditions. The novel GC risk prediction rule comprised five variables (age, sex, PG I/II ratio, G-17 level, and H. pylori infection), with scores ranging from 0 to 25. The observed prevalence rates of GC in the derivation cohort in the low-risk (0–11), medium-risk (12–16), and high-risk (17–25) groups were 1.2%, 4.4% and 12.3%, respectively. The developed and validated prediction rule showed good performance in identifying individuals at a higher risk in a Chinese high-risk population [21, 22].

In this study, we conducted a retrospective analysis of the data of patients who visited our hospital and met the novel scoring system and screening procedure criteria for GC screening, with an aim to explore the clinical application value of the novel scoring system for GC opportunistic screening.

## Materials and methods

### Study population

The study population included patients who visited our hospital from April 2017 to December 2019 for various digestive symptoms or health examinations and who completed gastroscopy (with biopsies), and serological tests, such as serum pepsinogen I/II (PG I/II), gastrin-17 (G-17), and anti-H. pylori IgG antibody (HP-IgG) tests.

The inclusion criteria included patients who were at least 40-years-old and met any of the following criteria: (1) residing in the areas of China with a high incidence of GC for more than 3 years; (2) a history of H. pylori infection; (3) a history of GC-related diseases such as chronic atrophic gastritis, gastric ulcer, gastric polyp, hypertrophic gastritis, and pernicious anaemia; (4) a positive family history of GC; (5) risk factors for GC, such as a high-salt diet, regular intake of pickled food, smoking, and heavy alcohol drinking.

The exclusion criteria included a definite history of GC or previous gastric resection.

The protocol was approved by the local ethics committee and was performed according to the Helsinki Declaration. All of the patients provided their written informed consent.

### Pathological diagnosis

Postoperative or gastroscopic biopsy pathology was defined as the gold standard for the final diagnosis. The final pathological diagnosis is determined by the dominant pathological type. Well-differentiated tubular adenocarcinoma, moderately differentiated tubular adenocarcinoma, and papillary adenocarcinoma were defined as differentiated gastric cancer (DGC). Poorly differentiated adenocarcinoma and signet-ring cell carcinoma were defined as undifferentiated gastric cancer (UDGC). High-grade intraepithelial neoplasia (HGIN), intramucosal carcinoma, and submucosal infiltrating carcinoma (irrespective of lymph node metastasis) were defined as EGC. Gastric atrophy (GA), intestinal metaplasia (IM), and low-level intraepithelial neoplasia (LGIN) were defined as precursors of gastric cancer (PGC).

### Study procedure

Relevant data of all of the enrolled patients were collected in chronological order to ensure continuity, including sex, age, gastroscopic and pathological results, and serological tests (PG I/II, G-17, and HP-IgG). All of the data were reviewed and confirmed by 2 or more senior endoscopic physicians and pathologists according to the standard protocol. All of the patients were divided into three groups according to the novel scoring system, which was established in the expert consensus opinion of the early gastric cancer screening project in China. Gastric cancer risk stratification according to total scores was as follows: 0–11 was regarded as low risk, 12–16 was regarded as medium risk, and 17–23 was regarded as high risk(Table 1) [21].

**Table 1:** The novel scoring system for GC screening

Variable	Classification	Score
Age(year)	40–49	0
	50–59	5
	60–69	6
	> 69	10
Sex	Female	0
	Male	4
HP-IgG	Negative	0
	Positive	1
PG I/II ratio	≥ 3.89	0
	< 3.89	3
G-17(pmol/L)	< 1.50	0
	1.50–5.70	3
	> 5.70	5

Three groups according to the score (0–23 points): Low-risk Group (0–11 scores), suggests low risk of GC; Medium-risk Group (12–16 scores), suggests some risk of GC; High-risk Group (17–23 scores), suggests high risk of GC

*GC* gastric cancer, *PG I/II* pepsinogen I/II, *G-17* gastrin-17, *HP-IgG* anti-H. pylori IgG antibody

### Statistical analysis

IBM SPSS Statistics for Windows (V.23.0) was used for the statistical analysis. The measurement data are shown as the mean (SD), and the counting data are shown in cases. The chi-square test was used to compare the detection rates of GC and PGC among the three groups. A two-sided P-value of less than 0.05 indicated statistical significance. The performance of the novel scoring system for GC opportunistic screening was assessed by the area under the curve (AUC) of the receiver operating characteristic curve (ROC).

## Results

### General condition

A total of 6,701 patients were enrolled in this study, including 4,352 patients (64.95%) in the low-risk group, 1,948 patients (29.07%) in the medium-risk group, and 401 patients (5.98%) in the high-risk group, according to the novel scoring system. Their mean (SD) age was 55.94 (10.04)-years-old, and 50.81% (3,405/6,701) of the patients were males.

### Detection of GC and PGC

A total of 190 cases of GC were detected in all 6,701 patients. The total detection rate of GC was 2.84% (190/6,701), with a 0.94% rate (41/4,352) in the low-risk group, a 5.18% rate (101/1,948) in the medium-risk group, and a 11.97% rate (48/401) in the high-risk group. There were statistically significant differences in the detection rate of GC among the three groups (all *P* < 0.05). In addition, the detection rates of PGC in the medium-risk group and high-risk group were significantly higher than those in the low-risk group(both *P* < 0.05), but there were no statistically significant differences between the medium-risk group and high-risk group (*P* > 0.05)(Table 2).

**Table 2:** Detection of GC and PGC by all risk group and score

Group/Score	Total No. (%)	GC No. (%)	GA/IM No. (%)	LGIN No. (%)
Low-risk	4352(64.95)	41(0.94)	1356(31.16)	40(0.92)
0	187(2.79)	0(0.00)	15(8.02)	0(0.00)
1	111(1.66)	0(0.00)	57(51.35)	0(0.00)
2	0(0.00)	0(0.00)	0(0.00)	0(0.00)
3	118(1.76)	2(1.69)	10(8.47)	1(0.85)
4	409(6.10)	3(0.73)	70(17.11)	2(0.49)
5	453(6.76)	1(0.22)	102(22.52)	2(0.44)
6	485(7.24)	3(0.62)	189(38.97)	4(0.82)
7	223(3.33)	2(0.90)	69(30.94)	2(0.90)
8	378(5.64)	1(0.26)	105(27.78)	7(1.85)
9	641(9.57)	3(0.47)	210(32.76)	7(1.09)
10	838(12.51)	14(1.67)	306(36.52)	10(1.19)
11	509(7.60)	12(2.36)	223(43.81)	5(0.98)
Medium-risk	1948(29.07)	101(5.18)^*^	723(37.11)^*^	36(1.85)^*^
12	293(4.37)	8(2.73)	110(37.54)	7(2.39)
13	395(5.89)	9(2.28)	152(38.48)	10(2.53)
14	405(6.04)	21(5.19)	156(38.52)	2(0.49)
15	492(7.34)	36(7.32)	157(31.91)	6(1.22)
16	363(5.42)	27(7.44)	148(40.77)	11(3.03)
High-risk	401(5.98)	48(11.97)^*#^	148(36.91)^*^	9(2.24)^*^
17	65(0.97)	3(4.62)	21(32.31)	1(1.54)
18	129(1.93)	19(14.73)	50(38.76)	5(3.88)
19	78(1.16)	4(5.13)	26(33.33)	1(1.28)
20	110(1.64)	14(12.73)	41(37.27)	1(0.91)
21	3(0.04)	1(33.33)	2(66.67)	0(0.00)
22	7(0.10)	2(28.57)	4(57.14)	1(14.29)
23	9(0.13)	5(55.56)	4(44.44)	0(0.00)
Total	6701(100.00)	190(2.84)	2227(33.23)	85(1.27)

*GC* gastric cancer, *PGC* precursors of gastric cancer, *GA* gastric atrophy, *IM* intestinal metaplasia, *LGIN* low-level intraepithelial neoplasia

^*^P < 0.05 vs. the Low-risk Group

^#^*P* < 0.05 vs. Medium-risk Group

Among the 190 GC cases, the detection rates of DGC in the medium-risk group (80.20%, 81/101) and high-risk group (81.25%, 39/48) were significantly higher than that in the low-risk group (58.54%, 24/41), according to the classification of differentiation (DGC and UDGC) (both *P* < 0.05). Specifically, the detection rate of UDGC in the low-risk group (41.46%, 17/41) was significantly higher than that in the medium-risk group (19.80%, 20/101) and high-risk group (18.75%, 9/48) (both *P* < 0.05). The detection rates of DGC and UDGC were not significantly different between the medium-risk group and the high-risk group(*P* > 0.05). In addition, there were no statistically significant differences in the detection rate of GC among the three groups according to the locations (cardia GC and noncardia GC) and the stages (EGC and AGC). Furthermore, the detection rate of EGC was 46.31% (88/190) among all of the detected GCs, and 73.86% (65/88) of them were detected in the medium-risk group and high-risk group (Table 3).

**Table 3:** Detection of different types of GC

Group	Total	EGC	AGC	DGC	UDGC	CGC	NCGC
Low-risk	41	23	18	24	17	5	36
Medium-risk	101	48	53	81*	20*	11	90
High-risk	48	17	31	39*	9*	11	37
Total	190	88	102	144	46	27	163

*GC* gastric cancer, *EGC* early gastric cancer, *AGC* advanced gastric cancer, *UDGC* undifferentiated gastric cancer, *DGC* differentiated gastric cancer, *CGC* cardia gastric cancer, *NCGC* non-cardia gastric cancer

^*^P < 0.05 vs. the Low-risk Group

### Predictive Value for the Risk of GC

**Fig. 1:**
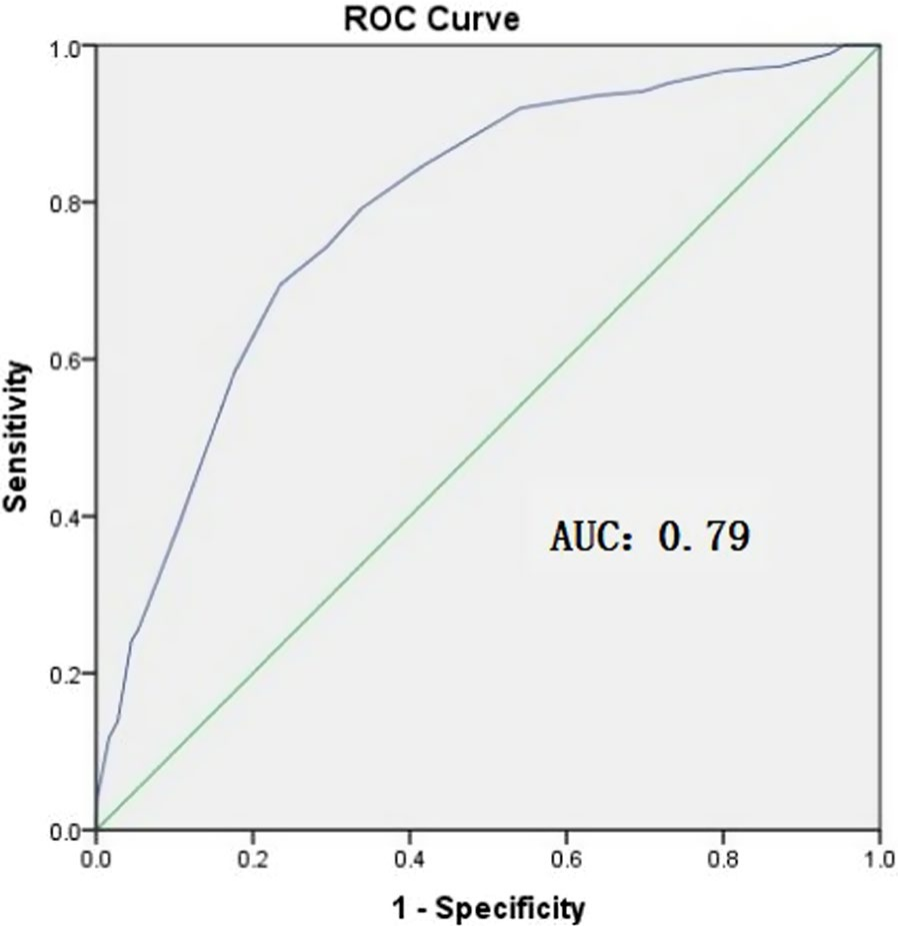
ROC of the Predictive Value for the Risk of GC

The predictive value of the novel scoring system for the risk of GC was investigated by ROC and the AUC was 0.79 (Fig. 1).

## Discussion

The development of tools for the early detection of GC is important for reducing mortality, increasing survival rates, and improving quality of life [23, 24]. Gastroscopy (with biopsies) is commonly accepted as the gold standard for detecting GC, but its application in the large-scale screening of GC is limited by its invasiveness and by an insufficient supply of endoscopy professionals and facilities [25, 26]. Therefore, it is urgent to develop novel, simple, cost-effective, and manipulable screening methods for GC.

In recent years, the serological test method has been widely used for EGC screening with the advantages of simplicity and noninvasiveness, and its effectiveness has been verified in large-scale research in China [27, 28]. A combination of the test of PG and HP-IgG, which is known as the ABC method, has been most widely used for GC screening [29–32]. However, one of its limitations is that Group A may include some individuals who have a high risk of AGC. GC was not detected in subjects without endoscopic atrophy, and the detection rates increased with the progression of atrophy [31]. In addition, the ABC method has been proven to be useful in urban and younger populations but is not applicable in populations with high prevalence of H. pylori infection and atrophic gastritis, whereas the latter is the high-risk population of GC and the focus of GC screening [32].

In this study, a novel scoring system that comprised five variables, including age, sex, HP-IgG, PG I/II ratio (PGR), and G-17 and which assigned different scores was used to provide an accurate risk stratification for GC opportunistic screening in hospital visits. The results indicated that there were statistically significant differences in the detection rate of GC among the three groups with different risk stratifications, and the detection rate of GC increased with the level of risk stratification. This suggests that the novel scoring system was indeed applicable to stratify the risk of GC and could be used for GC opportunistic screening in hospital visits. In addition, most (78.42%, 149/190) GC cases were detected in the medium-risk group and high-risk group. Therefore, gastroscopy should be strongly recommended for patients in these two groups.

The detection rate of EGC was 46.31% (88/190) among all of the GC cases in this study, which was significantly higher than the average detection rate of EGC in China [15]. This finding suggests that the screening strategy based on the scoring system and screening procedure was helpful to improve the detection rate of EGC. In addition, most (73.86%, 65/88) EGC cases were detected in the medium-risk group and high-risk group. Therefore, detailed gastroscopy should be adopted for patients in these two groups as much as possible to avoid missing the diagnosis of EGC.

DGC (which is equivalent to intestinal-type gastric adenocarcinoma) develops in a stepwise manner, with a sequence of events that progresses from inflammation to PGC (AG, IM, and LGIN) and GC, which is known as the Correa cascade. In this study, we found that the detection rates of PGC and DGC in the medium-risk group and high-risk group were significantly higher than that in the low-risk group. This finding indicated that the novel scoring system was also applicable to stratify the incidence of PGC, which suggests a high risk of DGC. This makes sense because the serological indicators used in this the novel scoring system are closely related to PGC [27, 28].

The detection rates of DGC in the medium-risk group (80.20%, 81/101) and high-risk group (81.25%, 39/48) were significantly higher than that in the low-risk group (58.54%, 24/41), according to the classification of differentiation (DGC and UDGC) (both P < 0.05). Specifically, the detection rate of UDGC in the low-risk group was significantly higher than that in the medium-risk group and high-risk group. It also suggested that the novel scoring system for GC opportunistic screening is only applicable to DGC, but not UGDC. Therefore, particular attention should be given to the possibility of UDGC for patients in the low-risk group. In addition, there were no statistically significant differences in the detection rate of GC among the three groups according to the locations (cardia GC and noncardia GC) and the stages (EGC and AGC). It also indicated that the novel scoring system for GC opportunistic screening could not be used to predict the location and stage of GC. Furthermore, the results of the ROC analysis showed that the AUC of the novel scoring system in differentiating GC is 0.79, thus indicating a good predictive value.

However, there were several potential limitations in the present study. First, the subjects who were included in this study were hospital visits, which are opportunistic screenings, and the results cannot represent the screening situation of the entire population. Second, all of the gastric lesions were only diagnosed from the biopsy specimens, which may make result in a small portion of GC being undiagnosed, thus resulting in a decrease in the detection rate of GC and the accuracy of the scoring system in predicting GC. Finally, this study was a retrospective and single-centre study, which may have resulted in some bias, thus further investigations using prospective study designs are required to evaluate the accuracy and discriminative ability of the scoring system.

## Conclusion

In conclusion, the screening strategy based on the novel scoring system and screening procedure can significantly improve the efficiency of GC opportunistic screening in hospital visits. Gastroscopy should be strongly recommended for patients in the medium-risk group and high-risk group, and detailed gastroscopy should be adopted as early as possible to improve the detection rate of EGC. Furthermore, appropriate follow-up strategies should be adopted for low-risk group to save on medical resources, for establishing and gradually improving the GC screening system in line with China's current national conditions.

## Data Availability

All the data supporting our findings are contained within the manuscript. The datasets used and/or analyzed during the current study are available from the corresponding author on reasonable request.

## Abbreviations

GC: Gastric cancer
AGC: Advanced gastric cancer
EGC: Early gastric cancer
PG I/II: Pepsinogen I/II
G-17: Gastrin-17
HP-IgG: Anti-H. pylori IgG antibody
DGC: Differentiated gastric cancer
UDGC: Undifferentiated gastric cancer
HGIN: High-grade intraepithelial neoplasia
GA: Gastric atrophy
IM: Intestinal metaplasia
LGIN: Low-level intraepithelial neoplasia
PGC: Precursors of gastric cancer
AUC: Area under the curve
ROC: Receiver operating characteristic curve
PGR: PG I/II ratio
